# Effects of Canopy Litter Removal on Canopy Structure, Understory Light and Vegetation Dynamics in *Cunninghamia lanceolata* Plantations of Varying Densities

**DOI:** 10.3390/plants14203144

**Published:** 2025-10-12

**Authors:** Lili Zhou, Lixian Zhang, Qi Liu, Yulong Chen, Zongming He, Shubin Li, Xiangqing Ma

**Affiliations:** 1 College of Geography and Oceanography, Minjiang University, Fuzhou 350108, China; fjzhoulili@126.com; 2 Research Institute of Subtropical Forestry, Chinese Academy of Forestry, Hangzhou 311400, China; 19559171995@163.com; 3 Forestry College, Fujian Agriculture and Forestry University, Fuzhou 350002, China; lllys021@126.com (Q.L.); hezm2@126.com (Z.H.); fjlishubin@126.com (S.L.); 4 College of Resources and Environment, Fujian Agriculture and Forestry University, Fuzhou 350002, China; cyylll1005@126.com

**Keywords:** marcescent species, canopy structure, light conditions, understory diversity, species richness, stand density, *Cunninghamia lanceolata*

## Abstract

The prolonged retention of senescent branches and needles (canopy litter) in *Cunninghamia lanceolata* canopies is an evolutionary adaptation, yet its impacts on stand microenvironment and understory succession remain poorly quantified. To address this gap, we conducted a 5-year field experiment across six planting densities (1800, 2400, 3000, 3600, 4200, and 4800 trees·ha^−1^), aiming to evaluate the effects of canopy litter removal on canopy structure, forest light environment, and understory biodiversity. Results demonstrated that leaf area index (LAI) and mean tilt angle of the leaf (MTA) significantly increased with density (*p* < 0.05), leading to marked reductions in photosynthetic photon flux density (PPFD) and light transmittance (T). Canopy litter removal significantly reduced LAI across all densities after 4–5 years (*p* < 0.05) and consistently enhanced PPFD and transmittance (*p* < 0.01). MTA and light quality parameters (red:blue and red:far-red ratios) both exhibited variable responses to litter removal, driven by density and time interactions, with effects diminishing over time. Understory vegetation diversity exhibited pronounced temporal dynamics and density-dependent responses to canopy litter removal, with increases in species richness (*S*), Simpson diversity (*D*), and Shannon–Wiener diversity (*H*), while Pielou Evenness (*J*) responded more variably. The most notable increase in species richness occurred in the 4th year, when 21 new species were recorded, largely due to the expansion of light-demanding bamboos (e.g., *Indocalamus tessellatus* and *Pleioblastus amarus*), heliophilic grasses (e.g., *Lophatherum gracile*) and pioneer ferns (e.g., *Pteris dispar* and *Microlepia hancei*). Correlation analyses confirmed PPFD as a key positive driver of all diversity indices (*p* < 0.01), whereas LAI was significantly negatively correlated with PPFD, light transmittance, and understory diversity (*p* < 0.01). These findings demonstrate that strategic management of canopy litter incorporating stand density regulation can improve understory light availability, thereby facilitating heliophilic species recruitment and biodiversity enhancement in subtropical coniferous plantations.

## 1. Introduction

Forest plantations have become an increasingly important component of global forest resources, playing a critical role in both wood production and ecological functions. The global area of forest plantations increased from 167.5 million ha in 1990 to 293.9 million ha in 2015, representing a rise from 4.06% to 7% of the total forest area [1,2]. In China, plantations cover approximately 69 million ha, accounting for about 25% of global plantation areas and ranking first in the world [3]. While the primary goal of these plantations has traditionally been timber and fuelwood production, ecological functions such as erosion control, carbon sequestration, and provision of environmental, economic, and social benefits have often been overlooked. In response, China has shifted its forest management strategy from prioritizing timber production to adopting multi-purpose plantation management that integrates broader social, economic, and environmental objectives [4]. However, intensive management practices, such as monocultures, short rotation cycles, and clear-cutting, have led to a simplified stand structure, reduced biodiversity, and degraded ecosystem services [5]. This raises the need for strategies to modify forest structure and enhance biodiversity to ensure long-term forest sustainability.

Chinese fir (*Cunninghamia lanceolata*) is a highly valued timber species with over a thousand years of cultivation history in China. It is distributed across 17 provinces, ranging from 19°30′ to 34°03′ N latitude and 101°30′ to 121°53′ E longitude [6]. Its plantation area is approximately 11 million ha, accounting for 15.8% of China’s total plantation area and 6.1% of the global forest plantation area [7]. One key characteristic of *C. lanceolata* is the prolonged retention of senescent branches and needles in the canopy, which remain for many years before abscission. The huge mass of canopy litter ranges from 14.2 to 17.4 t·ha^−1^ across different developmental stages [8,9]. Once canopy closure occurs in Chinese fir plantations, the lower branches and needles gradually die due to limited light conditions and remain in the canopy, comprising approximately 95% of the total aboveground litter (including the litterfall on the forest floor) [10]. The long-term retention and accumulation of these dead branches and needles alter canopy structure, affect light penetration, and ultimately influence the growth and succession of understory communities [11]. Previous studies have largely focused on the spatiotemporal distribution, decay rates, and nutrient dynamics of canopy litter [8,12,13]. However, the role of persistent canopy litter in mediating overstory structure and its cascading effects on understory light availability and subsequent vegetation recruitment remains insufficiently explored.

A diverse and well-developed understory plays a crucial role in maintaining forest functional stability, enhancing ecosystem resilience, and supporting various ecological processes [14]. Understory vegetation is strongly influenced by overstory canopy structure and understory light availability [15,16]. Leaf area index (LAI) and mean tilt angle of leaves (MTA) are important canopy structure traits. LAI quantifies the total leaf area per unit of ground area (m^2^·m^−2^), and MTA refers to the angle at which leaves are oriented relative to the ground (°). Higher LAI and lower MTA typically reduce light penetration to the forest floor [17], thus promoting the growth of shade-tolerant species [18]. Increasing LAI enhances canopy light interception and causes a sharp decline in understory photosynthetic photon flux density (PPFD), a pattern typically characterized by an exponential decay function [19]. In contrast, canopies with more horizontally oriented leaves facilitate greater light penetration to understory, thereby modifying the vertical distribution of PAR and PPFD and influencing understory species composition and regeneration dynamics [20,21].

These variations in understory PPFD directly impact light capture efficiency, crown morphology, and recruitment of seedlings and understory plants [22]. Reduced light intensity at the forest floor limits the growth of light-demanding species, while favoring shade-tolerant species [23]. Brenes-Arguedas et al. [24] found that understory light availability influences the growth and distribution of shade-tolerant seedlings, although the magnitude and direction of these effects were strongly modulated by water availability along a tropical rainfall gradient. Light quality also plays an important role in the development of understory species. In particular, the red:blue and red:far-red ratios can enhance both growth and antioxidant capacities in herbs compared with white light or sunlight [25]. Similarly, in temperate forests, small-seeded herb species require higher red:far-red ratios for successful germination [26]. Therefore, understanding how overstory canopy structure modulates understory light availability (including both quantity and quality) and subsequent vegetation dynamics is essential for improving ecological restoration strategies in plantations worldwide.

In this study, we hypothesized that: (1) The canopy litter removal will significantly alter overstory canopy structure (e.g., LAI and MTA) and understory light conditions (e.g., PPFD, light transmittance, red:blue ratio, and red:far-red ratio), with these effects varying over time. (2) The canopy litter removal will increase understory plant diversity, with differential impacts on the shrub and herb layers, and these effects will be more pronounced in the short term than in the long term. To test these hypotheses, we investigated overstory canopy structure, understory light environment, and species composition and diversity in the understory layer under canopy litter removal and retention treatments across different planting densities during 2–5 years in Chinese fir plantations. Additionally, we examined the relationships between canopy structure, understory PPFD, and understory diversity. Our results will contribute to a better understanding of how canopy litter influences forest light environments and understory vegetation dynamics and provide valuable insights for enhancing the sustainability and biodiversity of Chinese fir plantations.

## 2. Results

### 2.1. Effects of Canopy Litter Removal on Overstory Canopy Structure

Three-way ANOVA results (Table 1) showed that canopy litter treatment, stand density, time, and their interactions significantly affected LAI and MTA of the forest stands, except for the interaction between canopy treatment and stand density on LAI and the main effect of canopy treatment on MTA (*p* < 0.01).

**Figure 1:**
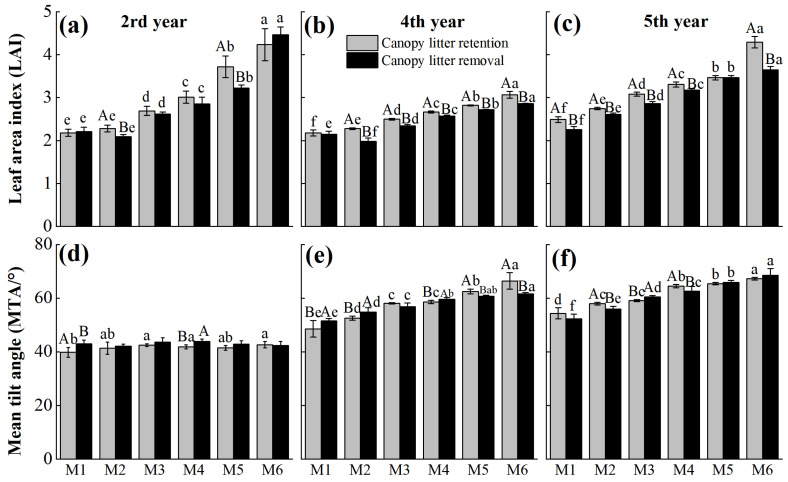
Effects of canopy litter removal on leaf area index (**a**–**c**) and mean tilt angle (**d**–**f**) at 2nd, 4th, and 5th year after treatment under different planting densities. M1, M2, M3, M4, and M5 represent planting densities of 1800, 2400, 3000, 3600, 4200, and 4800 stems·ha^−1^, respectively. Vertical bars are mean values ± SD (*n* = 3). Different uppercase letters indicate significant differences between the canopy litter retention and removal at the same density and time point (*p* < 0.05). Different lowercase letters indicate significant differences among planting densities under the same canopy litter treatment at each time (*p* < 0.05). Letters are shown only where significant differences occur.

The LAI and MTA generally increased with increased stand density (except for the MTA in the 2nd year of post-treatment) (Figure 1). Canopy litter removal led to varying decreases in LAI across different stand densities in the 2nd, 4th, and 5th years, except for M1 in the 2nd year (Figure 1a–c). Significant differences in LAI between treatments at each density were observed in the 4th and 5th years (except for M1 in the 4th year and M5 in the 5th year) (*p* < 0.05). In contrast, MTA was higher than the control in all densities two years after canopy litter removal. By the 4th year, MTA significantly increased under densities M1, M2, and M4, but decreased under M5 and M6. Five years after treatment, MTA showed a reversed pattern compared to the 4th year (Figure 1d–f). These results suggest that LAI and MTA responses to canopy litter removal are density-dependent and time-accumulative.

### 2.2. Effects of Canopy Litter Removal on Understory Light

Three-way ANOVA results (Table 2) showed that canopy litter treatment, stand density, time, and their interactions had significant effects on PPFD and light transmittance (*p* < 0.001). Stand density, time, their interaction, and the three-way interaction among all factors significantly affected on the red/blue and red/far-red ratio (*p* < 0.01). However, canopy litter treatment had no significant effect on either spectral ratio.

**Figure 2:**
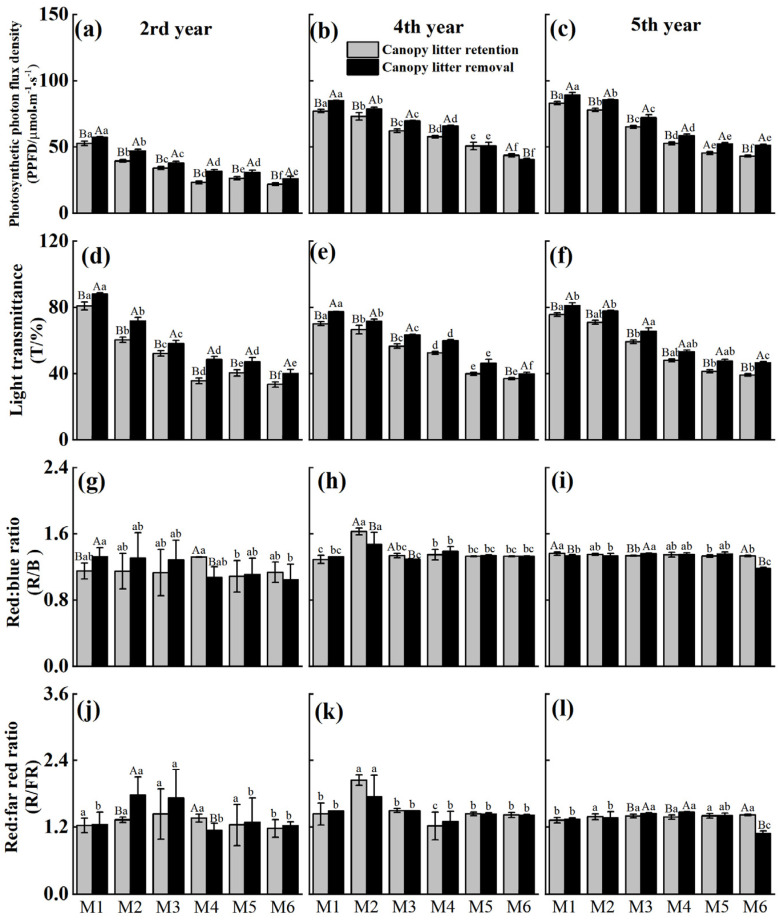
Effects of canopy litter removal on photosynthetic photon flux density (**a**–**c**), light transmittance (**d**–**f**), red:blue ratio (**g**–**i**) and red:far-red ratio (**j**–**l**) at 2nd, 4th, and 5th year after treatment under different planting densities. M1, M2, M3, M4, and M5 represent 1800, 2400, 3000, 3600, 4200, and 4800 stems·ha^−1^, respectively. Vertical bars are mean values ± SD (*n* = 3). Different uppercase letters indicate significant differences between the canopy litter retention and removal at the same density and time point (*p* < 0.05). Different lowercase letters indicate significant differences among planting densities under the same canopy litter treatment at each time (*p* < 0.05). Letters are shown only where significant differences occur.

PPFD and light transmittance within the forest significantly decreased with increasing stand density. For both canopy litter removal and retention treatments, the highest-density stand (M6) exhibited approximately 50% reduction in both PPFD and light transmittance compared to the lowest-density stand (M1) (Figure 2a–f). Across most densities and time points, canopy litter removal significantly increased PPFD and transmittance compared to the litter-retained treatment, except for PPFD in M5 and M6 and transmittance in M4 and M5 during the 4th year.

The effects of canopy litter removal on light quality varied with stand density and gradually diminished over time (Figure 2g–l). Two years post-treatment, the red/blue and red/far-red ratios increased in low- to medium-density stands (M1–M3), while changes were minimal in high-density stands. By the 4th year, there were no significant differences in light quality between treatments at most densities, except for M2. By the 5th year, the red/blue and red/far-red ratios were significantly higher in M3 under canopy litter removal, whereas in M6, these ratios were significantly lower under canopy litter removal compared with the retention treatment.

### 2.3. Effects of Canopy Litter Removal on Understory Vegetation

#### 2.3.1. Understory Composition and IVI

Canopy litter removal had a significant short-term effect on increasing understory species richness (*S*). A slight increase was observed in the second year (81 species in removal vs. 78 species in retention), with the peak occurred in the 4th year (85 species in removal vs. 64 species in retention), representing an addition of 21 species. However, this effect diminished by the 5th year (73 species in removal vs. 72 species in retention), indicating a clear ecological time lag in the understory response to litter removal. Specifically, canopy litter removal significantly affected understory species richness with clear layer-specific, temporal, and density-dependent effects. Among all the subplots, herbaceous species richness (average 62 species) was significantly higher than shrub species richness (average 13 species) (Table 3). Canopy litter removal significantly promoted herbaceous richness (from 58 species in retention treatment to 67 species in removal treatment), but had no significant overall impact on shrubs, with complex interannual fluctuations (Table 3). Compared with the retention treatment, herbaceous species richness showed a minimal increase of 2 species in the 2nd year, peaked at an enhancement of 16 species in the 4th year and declined to 10 species by the 5th year, reflecting a short-term but pronounced response in understory dynamics.

Treatment effects varied strongly with stand density (Table S1). In high-density stands (M6), understory vegetation was sparse, with only 2–3 shrub species and 21–25 herbaceous species in retention treatment. Canopy litter removal increased herbaceous species to 25–50 species with a peak in the 4th year. In low- to medium-density stands (M1-M3), retention treatment already had a relatively rich herb layer (26–34 species), and canopy litter removal further significantly increased herb species (peaking at 59–72 species in the 4th year), surpassing the high-density stands in terms of richness. Shrub species showed no consistent response across densities.

The five most abundant species in the shrub and herb layers at 2, 4, and 5 y post-removal were listed in Tables S2–S4. Although shrub species richness was low, canopy litter removal greatly promoted the expansion of light-demanding bamboo species. In the second year, early positive succession with the emergence of light-demanding pioneer *Callicarpa kochiana* and became dominant in M2. The temporary appearance of *Lithocarpus glaber* and *Schima superba* further indicated that canopy litter removal stimulated forest regeneration. By the 4th year, some heliophilous shrubs such as *Premna microphylla*, *Ampelopsis cantoniensis*, and *Syzygium grijsii* expanded. Meanwhile, shade-tolerant species such as *Maesa japonica* and *Eurya nitida* began to decline. In the 5th year, the light-demanding bamboo such as *Pleioblastus amarus and Indocalamus latifolius* ranked highest in importance values. These findings indicate that canopy litter removal improved understory light and strongly facilitated the clonal expansion of bamboo species.

Across all stand densities, the herbaceous layer was initially dominated by shade-tolerant fern species such as *Woodwardia japonica*, *Lindsaea orbiculata*, and *Allantodia metteniana*. However, the dominance of these species declined over time. The IVI of *Lindsaea orbiculata* decreased markedly from 90.1% in the 2nd year to 18.9% in the 5th year, reflecting reduced competitiveness under increased light (Tables S2–S4). Conversely, the IVI of light-demanding herbaceous species increased markedly. Graminaceous species such as *Lophatherum gracile*, *Cyrtococcum patens*, and *Oplismenus undulatifolius* showed pronounced increases in the 4–5 years, particularly in high-density stands (M4–M6). Similarly, pioneer heliophilous fern species, including *Pteris dispar* and *Microlepia hancei*, also became more abundant. Overall, the herbaceous layer in Chinese fir plantations is naturally dominated by shade-tolerant ferns. However, canopy litter removal greatly improved the light environment, thereby promoting niche differentiation and species turnover. This shift progressively favored heliophilous grasses and pioneer ferns, with light-driven community restructuring becoming more pronounced over time.

#### 2.3.2. Understory Vegetation Diversity

Three-way ANOVA indicated that canopy litter treatment, stand density, time, and their interactions significantly influenced understory vegetation diversity (Table 4). Simpson’s index was significantly affected by treatment, density, and the treatment × time interaction. Shannon–Wiener index was significantly influenced by treatment, density, and the interactions of treatment × density, treatment × time, and density × time. Pielou’s evenness index was significantly affected by all factors except for the treatment. All factors, except for the treatment × density interaction and the three-way interaction significantly, impacted the diversity of oddness index.

**Figure 3:**
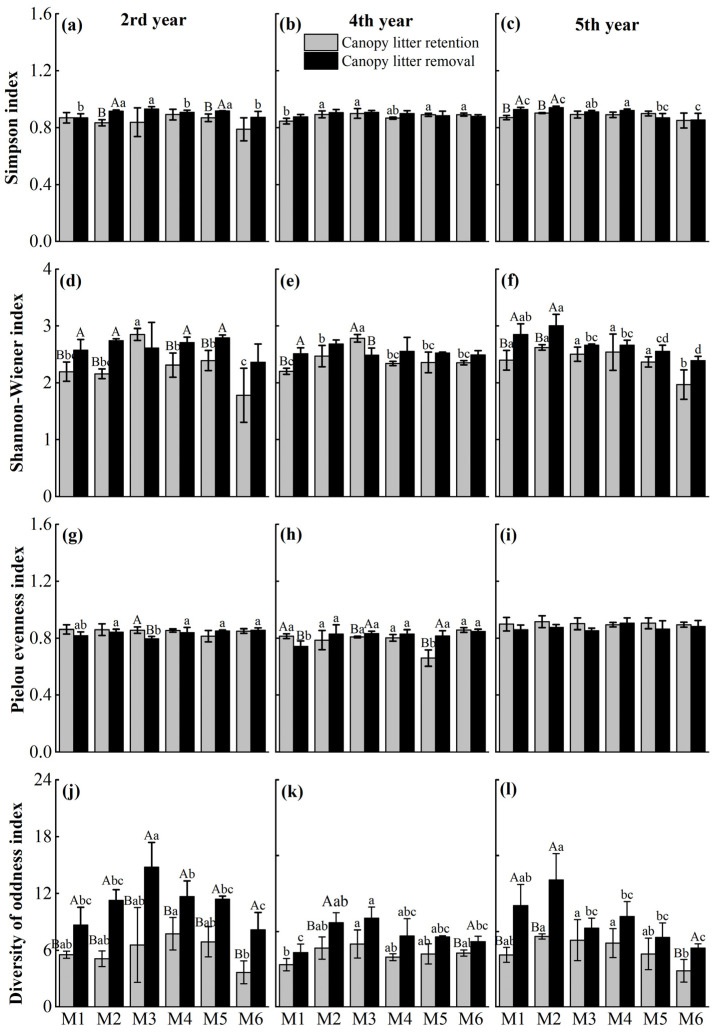
Effects of canopy litter removal on Simpson index (**a**–**c**), Shannon–Wiener index (**d**–**f**), Pielou evenness index (**g**–**i**), and diversity of oddness index (**j**–**l**) at 2nd, 4th, and 5th year after treatment under different planting densities. M1, M2, M3, M4, and M5 represent planting densities of 1800, 2400, 3000, 3600, 4200, and 4800 stems·ha^−1^, respectively. Vertical bars are mean values ± SD (*n* = 3). Different uppercase letters indicate significant differences between the canopy litter retention and removal at the same density and time point (*p* < 0.05). Different lowercase letters indicate significant differences among planting densities under the same canopy litter treatment at each time (*p* < 0.05). Letters are shown only where significant differences occur.

Canopy litter removal significantly increased community dominance and diversity, though reducing species evenness (Figure 3). Compared to retention treatment, Simpson’s diversity generally increased in removal stands, except at M1 in the 2nd year, M5–M6 in the 4th year, and M5 in the 5th year. Shannon–Wiener diversity also increased broadly, except at M3 in the 2nd and 4th years, indicating enhanced richness and compositional complexity. Pielou’s evenness decreased at M1–M4 in the 2nd year and at M1–M4 and M6 in the 5th year, but increased at M2–M5 in the 4th year, reflecting temporal variability in community balance. The diversity of oddness index remained higher in treated plots across all years, with significant increases (*p* < 0.05) across densities in the 2nd year, highlighting early positive effects on recruitment of rare species.

### 2.4. Relationships Between Overstory Canopy Structure, Understory Light and Understory Vegetation

#### 2.4.1. Relationships Between Overstory Canopy Structure and Understory Light

**Figure 4:**
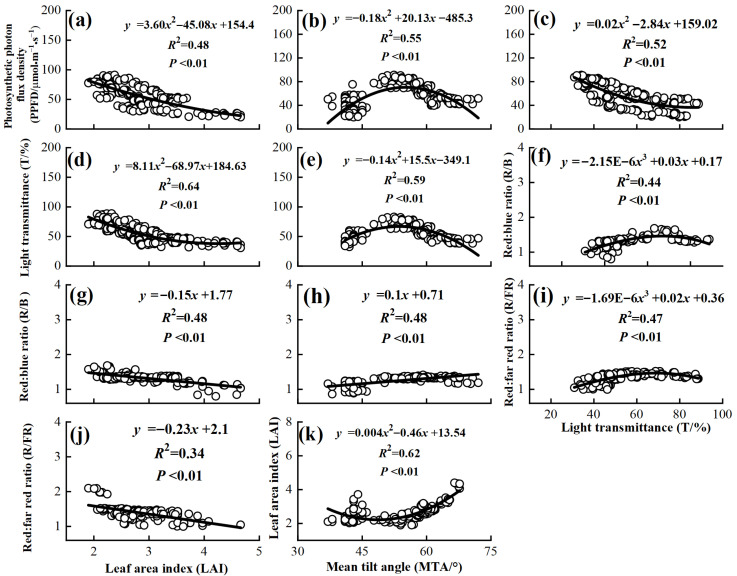
Fitted relationships between leaf area index and photosynthetic photon flux density, light transmittance, red:blue ratio and red:far red ratio (**a**,**d**,**g**,**j**), between mean tilt angle and photosynthetic photon flux density, light transmittance, red:blue ratio and leaf area index (**b**,**e**,**h**,**k**), and between light transmittance and photosynthetic photon flux density, red:blue ratio, and red:far red ratio (**c**,**f**,**i**).

Overstory canopy structure was closely related to the understory light environment (Table 5, Figure 4). LAI was significantly positive with MTA and the red/far-red ratio (*p* < 0.01) but significantly negative with PPFD, light transmittance, and the red/blue ratio. MTA showed significant positive correlations with PPFD and red:blue ratios but a significant negative correlation with light transmittance. PPFD and light transmittance were significantly positively correlated with both red:blue and red:far-red ratios. These results indicate that increased LAI reduces PPFD and light transmittance, while enhancing red/far-red light enrichment. Conversely, higher light transmittance improves both light intensity and spectral quality, potentially stimulating the growth of heliophilous species.

#### 2.4.2. Effects of Overstory Canopy Structure, Understory Light on Understory Vegetation

Correlation analysis (Table 6) showed that PPFD and light transmittance were highly positively correlated with Simpson diversity, Shannon–Wiener, and the diversity of oddness index (*p* < 0.01). Both red/blue and red/far-red light ratios synergistically promoted the stability of functional diversity groups, with strong positive correlations with Simpson’s index and diversity of oddness index. In contrast, LAI negatively impacted understory species diversity, showing significant negative correlations with all diversity indices (*p* < 0.01), indicating that dense canopy constrains species coexistence. MTA had no direct effect on species richness, with its influence mediated by light conditions. Species evenness increased significantly with higher LAI and showed a non-significant decrease with higher light intensity and spectral ratios.

## 3. Discussion

### 3.1. Effects of Canopy Litter Removal on Overstory Structure and Understory Light Environment

Our results supported the first hypothesis that canopy litter removal significantly improved the understory light environment by modifying canopy structure. Across all stand densities, LAI decreased continuously during 2–5 years after treatment, reaching significant differences in years 4–5 (*p* < 0.05; Table 1, Figure 1), leading to significantly increased understory PPFD (Table 2, Figure 2). LAI was significantly negatively correlated with PPFD and light transmittance (Table 5, Figure 4), confirming the key role of canopy structure in regulating understory light dynamics [27]. Dense canopies enhance light interception and reduce PPFD transmission to the understory by overlapping branches [28], a process closely related to leaf lifespan and MTA. *Cunninghamia lanceolata* exhibits high marcescent litter retention and slow decomposition rates, with marcescent litter biomass in young to mature stands reaching 14.2–17.4 t·ha^−1^ [8]. Prolonged retention in the canopy substantially increases LAI, whereas marcescent litter removal effectively reduced LAI across stand densities, thereby increasing understory total PPFD.

In contrast, the effects of canopy litter removal on MTA showed complex fluctuations depending on stand density and time. In the 2nd and 4th year after removal, MTA increased in low-density stands (M1 and M4) but decreased in the high-density stand (M6); by the 5th year, this pattern reversed (Figure 1). This indicates that marcescent litter not only affects the absolute leaf area but also the spatial distribution of leaves, thereby affecting the heterogeneity of understory light. In low-density stands, improved light conditions following canopy litter removal induced an increase in MTA, whereas in high-density stands, intense light competition limited the effects of litter removal on light regulation, resulting in negligible or even reduced changes in MTA [29].

Canopy litter retention increased canopy closure, which not only directly reduces understory PPFD but also significantly affects light quality [30]. In this study, differences in the red/blue and red/far-red ratios among treatments also exhibited significant interactions between stand density and time, with effects diminishing over time. In the 2nd year, canopy litter removal increased R/B and R/FR in low-density stands, consistent with the findings of Leuchner et al. [31] in mixed *Picea abies–Fagus sylvatica* forests in southern Germany. In dense stands, increased LAI substantially reduced the proportion of direct radiation reaching the understory, where transmitted light was dominated by diffuse or scattered radiation [32]. Due to its shorter wavelength, blue light is more susceptible to absorption and scattering by leaves, leading to pronounced attenuation under dense canopies. In contrast, the longer wavelength of far-red light makes it less susceptible to absorption, thus leading to higher far-red irradiance and lower R/FR ratios beneath the canopy [33]. Canopy litter removal increased canopy openness and enhanced light intensity, while progressively diminishing differences in light quality among treatments.

In addition, both canopy structure and the light environment of *Cunninghamia lanceolata* stands exhibited pronounced density-dependent effects. As stand density increased, LAI rose from 2.28 in M1 to 3.86 in M6, while MTA increased from 47.58°to 58.76°(Figure 2). The synchronous increase in LAI and MTA suggests that high-density stands enhance light interception capacity by both increasing leaf area and adjusting leaf orientation toward a more horizontal distribution [34,35,36]. Meanwhile, understory light transmittance declined sharply from 75.53% to 36.50% (Figure 2d–f), indicating strong suppression of light penetration by a closed canopy. The presence of marcescent litter further intensified this density-driven shading effect. Beyond mechanically blocking light, it likely altered the spectral composition and microclimatic conditions beneath the canopy, thereby influencing understory plant growth and development [37,38].

### 3.2. Effects of Canopy Litter Removal on Understory Vegetation Composition and Diversity

Our results partially supported the second hypothesis, indicating that canopy litter removal induced a short-term increase in understory plant species diversity. The peak in newly recorded species (21 species) occurred in the 4th year after treatment, when total species richness reached its maximum (85 species). This finding suggests that enhanced light availability can activate the soil seed bank and facilitate the colonization of understory plants. Previous studies have also reported that canopy disturbance stimulated the emergence of early-successional species and accelerated community renewal [39]. However, such species increment was not evident in the 2nd year, showing a pronounced ecological time lag, which is primarily attributed to the 1–3 years required for species to progress from seed germination to seedling establishment [40]. In our study, canopy litter removal also resulted in substantial accumulation of senescent branches and needles on the forest floor. The thick litter layer likely acted as a physical barrier, potentially inhibiting seed germination and seedling establishment. Consequently, although canopy litter removal improved understory light availability and temporarily increased species diversity, the excessive litter accumulation may have partially offset these positive benefits. While this study focused primarily on how canopy litter removal influences light environment and understory community dynamics, the broader ecological consequences of litter redistribution for understory biodiversity warrant further investigation.

In addition, we found that the effects of litter removal on understory vegetation were clearly stratified. Herb layer abundance showed a strong short-term response; by the 4th year, the treatment increased herbaceous species by 16, whereas shrub species increased by only 5. This pattern is closely related to the light competition advantage and rapid life-history strategies of the herb layer [41]. The improved light conditions and activation of the seed bank promoted the invasion of new species, with herbaceous plants showing a clear competitive advantage over shrubs [23,42]. In untreated stands, shade-tolerant ferns (e.g., *Woodwardia japonica*) were dominant. This aligns with our findings that higher LAI favors the homogenized distribution of shade-tolerant ferns like *Woodwardia japonica*, while improved light conditions promote the proliferation of bamboo and pioneer heliophilous species, which increases diversity but reduces evenness. After canopy litter removal, increased light availability facilitated the expansion of bamboo species (*Indocalamus tessellatus* and *Pleioblastus amarus*) and the emergence of heliophilous herbaceous species (*Lophatherum gracile*, *Cyrtococcum patens*, etc.). This indicates that litter removal altered the competitive structure of the community by improving light conditions and the microenvironment, but also promoted a functional shift from shade-tolerant to light-demanding communities [39,43].

The response of understory vegetation to canopy litter removal was strongly influenced by stand density. In low- to medium-density stands (M1–M3), higher initial canopy openness and baseline richness allowed litter removal to further increase both PPFD and species richness. In contrast, in high-density stands (M5–M6), initial understory diversity was low, and canopy litter removal primarily promoted the expansion of strong light competitors such as bamboo and the decline of shade-tolerant ferns in the 4th year, while opportunities for new species establishment remained limited. For example, canopy litter removal in M6 added 29 herbaceous species, compared with 39 in M3 (Table S1). This highlights the critical role of stand density in determining the biodiversity-enhancing effects of canopy litter removal [44,45]. Moreover, over time, intensified competition among light-demanding species and monopolization by dominant taxa led to a gradual homogenization of the community and a decline in evenness [46,47], consistent with the findings of Cazzolla et al., [48]. These dynamics indicate that canopy litter removal regulates understory light conditions and drives dynamic changes in species composition, functional group structure, and diversity levels, but its facilitative effects are clearly both stage-specific and density-dependent [49]. In commercial Chinese fir plantations, moderate stand densities (similar to M2–M3) are generally preferred to optimize both timber production and ecological functions. For high-density stands, our results suggest that integrating occasional selective thinning with targeted canopy litter management can improve stand microenvironment, thereby facilitating the recovery and diversity of understory species.

### 3.3. Implications for Forest Management

Our findings demonstrate that canopy litter removal can serve as an effective silvicultural practice for enhancing the understory light environment and the composition and diversity of understory communities, particularly in low- and moderate-density *C*. *lanceolata* plantations. In high-density stands, however, the benefits derived from improved light conditions were more limited, suggesting that management strategies should explicitly account for the influence of stand density and canopy structure when implementing canopy litter management. Canopy litter removal effectively complements conventional silvicultural practices such as thinning and pruning by enhancing light availability and moderating the understory microclimate. Moreover, pruning promotes stem straightness and reduces knot formation. To cultivate large-diameter and knot-free timber, we recommend initiating moderate pruning at young and middle-aged stages, with pruning intensity flexibly adjusted according to stand density, tree age, and site conditions. Therefore, integrating moderate stand density, periodic pruning, selective thinning, and targeted canopy litter removal may provide a practical strategy for balancing timber production, wood quality and biodiversity conservation in subtropical conifer plantations [50].

## 4. Materials and Methods

### 4.1. Study Site and Experimental Design

**Figure 5:**
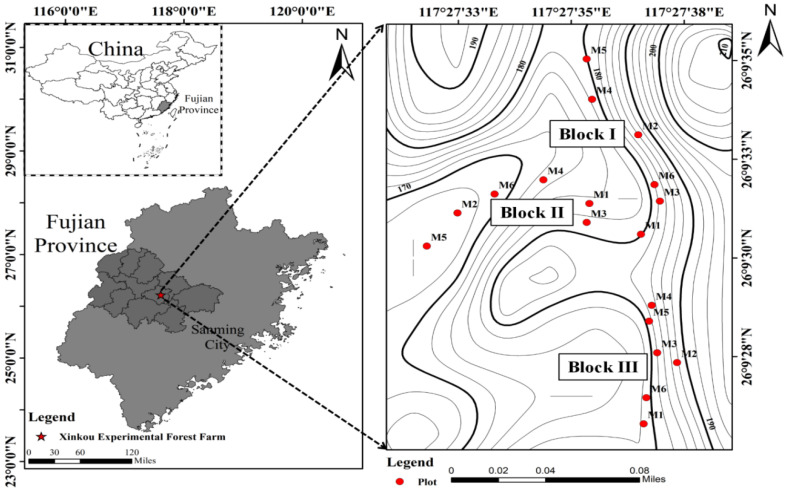
Location of the experimental Chinese fir plots in Sanming city, Fujian province, China. M refers to the stand densities detailed in Table 1. Light gray represents Fujian Province, and dark gray represents Fuzhou City.

This study was conducted at the Xinkou Experimental Forest Farm (26°07′–27°13′ N, 117° 27′–118°14′ E; 205–500 m a.s.l.), located in Sanming City, Fujian Province, South China (Figure 5). The site experiences a typical subtropical marine monsoon climate, with a mean annual temperature of 19.1 °C. The mean monthly maximum and minimum temperature are 28.8 °C in July and 10.4 °C in January, respectively. The average annual rainfall is 1749 mm, with the majority of precipitation (48%) occurring from April to June. Mean annual sunshine duration is 1840 h, and the frost-free period extends 300 days per year, with frost occurring exclusively in January and February [51]. According to the U.S. taxonomy system, the soil was Silty Oxisol, developed from sandstone and shale parent materials.

The Chinese fir stands were established in 2007 using one-year-old seedlings, following clear-cutting and the burning of harvest residuals at an elevation of approximately 210 m a.s.l. A randomized complete block design was implemented with three replicate blocks, each containing six 20 × 20 m plots (18 plots in total). The experimental planting densities were 1800 stems·ha^−1^ (M1, 2.36 × 2.36 m spacing), 2400 stems·ha^−1^ (M2, 2.04 × 2.04 m spacing), 3000 stems·ha^−1^ (M3, 1.83 × 1.83 m spacing), 3600 stems·ha^−1^ (M4, 1.67 × 1.67 m spacing), 4200 stems·ha^−1^ (M5, 1.54 × 1.54 m spacing), and 4800 stems·ha^−1^ (M6, 1.44 × 1.44 m spacing). The afforestation was carried out on land previously occupied by Chinese fir plantations. Seedlings sourced from a second-generation Chinese fir seed orchard were manually planted in hand-dug holes. In each stand, weeding was conducted twice annually (June and September) in the first 3 years and then once every 4 years thereafter. Replanting was performed as needed.

Pre-treatment measurements of diameter at breast height (DBH) and tree height were conducted in all plots during December 2018. The characteristics for the different density plantations prior to the canopy litter treatments are presented in Table 7. The canopy litter removal experiment was established in the spring of 2019, when the plantations of different densities were 12 years old. At this stage, the stands had accumulated a substantial amount of retained canopy litter, with no thinning or pruning having been implemented. Canopy litter was removed using a combination of pole pruners and machetes, and all removed litter was left within plots to maintain the site’s natural biomass balance. Each density plot within every block was divided into four equal 10 × 10 m subplots. Two independent subplots were randomly selected and assigned to either canopy litter removal or retention. To ensure physical isolation between treatments, subplots were prioritized along diagonal positions. During field surveys, a 1–2 m peripheral belt within each subplot was designated as a buffer zone to minimize edge effects, while the central 8 × 8 m core sampling area for ecological measurements. In total, 36 independent experimental subplots were obtained (3 blocks × 6 densities × 2 canopy litter treatments).

### 4.2. Measurements of Overstory Canopy Structure and Understory Light

Overstory canopy structure and understory light conditions were assessed regularly in the second (2021), fourth (2023), and fifth (2024) years after canopy litter removal treatments in both May and September. Seasonal values were averaged to represent annual means. Canopy structure was measured between 08:00 and 10:00 under uniformly overcast conditions to minimize solar glare and ensure consistent diffuse radiation [17]. A canopy analyzer (Yaxin-1201, Beijing, China) was used for these measurements. Initially, six hemispherical photographs were taken outside the forest as a calibration reference. Then, in each subplot, 12 random sampling points were selected, and three hemispherical photographs were taken at 1.5 m height with vertical lens orientation at each point, resulting in 36 photos per subplot (1296 photos in total for the 36 subplots). Images were batch-processed using Yaxin-1201 software (Version 1.3.1) with threshold segmentation applied to distinguish canopy elements from the sky. Leaf area index (LAI) and mean tilt angle of leaves (MTA) were extracted as key canopy structural indicators.

Understory light was measured between 09:00 and 12:00 on overcast or misty days with stable irradiance, ensuring that the light intensity remained above the sensor’s detection threshold. Before entering the forest, exterior photosynthetic photon flux density (PPFD, μmol·m^−2^·s^−1^) was measured at six randomized points outside the forest using a portable light quantum flux sensor (HP3500, Taiwan, China). Within each subplot, twelve sampling points were established to record PPFD and spectral bands (red, 655–665 nm; far-red, 725–735 nm; blue, 450–470 nm). At each point, three replicate readings were taken and averaged. Light transmittance (*T*) was then calculated using the following formula [52]:

(1) $$T\;=\;\frac{{\mathrm{PPFD}}_{\mathrm{understory}}}{{\mathrm{PPFD}}_{\mathrm{outside}}}\;\times \;100\%$$

where PPFD_understory_ is the PPFD inside the forest canopy and PPFD_outside_ is the PPFD measured outside the forest.

### 4.3. Understory Surveys and Diversity Calculations

Floristics surveys of understory vegetation were conducted in October of the 2nd year (2021), 4th year (2023), and 5th year (2024) after the canopy litter removal treatment. In each 10 × 10 m subplot, five 2 × 2 m quadrats for shrubs and five 1 × 1 m quadrats for herbs were randomly selected in a “Z” pattern. Vegetation was classified into shrub and herbaceous layers, with shrubs defined as individuals 1 m < height ≤ 3 m and herbs as non-woody species < 0.5 m in height. Data from the five small quadrats for each layer (shrub and herb) were combined to characterize the subplot.

Species were identified using the eFlora of China database (http://www.cn-flora.ac.cn/; accessed on 8 July 2025). For both shrub and herb layers, species identity and their numbers were recorded to calculated relative density. For shrubs, coverage, frequency, height, and number of individuals were recorded. Due to the difficulty in measuring height for the herbaceous layer, coverage was measured for each herb species instead. For clonal herb species, each clone unit was treated as a single individual.

To describe species composition, the importance value index (IVI) was calculated as the average of relative density (*R*_de_), relative dominance (*R*_do_), and relative frequency (*R*_f_). *R*_do_ was stratum-specific, with shrub-layer values derived from cumulative height measurements, while herb-layer values were based on vegetation coverage assessments. The theoretical range for *R*_do_, *R*_f_, *R*_d_ and *R*_c_ is 0~100%, so the IVI also falls within this range, because each IVI is the average of these three percentages. Key parameters are defined as follows:


Relative density (Rde) = number of individuals of a species/total number of individuals × 100
(2)

Relative dominance (Rdo) = total height or coverage of a species/total height or coverage of all species × 100
(3)

Relative frequency (Rf) = frequency of a species/total frequencies of all species × 100
(4)

Five indices were chosen to evaluate understory species diversity, and their definitions and formulas are as follows [53,54]:


Richness (S): total number of species recorded per treatment or density
(5)

Shannon–Wiener diversity index (*H*):

(6) $$H=-\sum_{i=1}^{S}P_{i}lnP_{i}$$

Simpson diversity index (*D*):

(7) $$D=1-\sum_{i=1}^{S}P_{i}^{2}$$

Evenness index (*J*):

(8) $$J=\frac{H}{lnS}$$

Diversity of oddness index (*OD*):

(9) $$OD={\left(\sum_{i=1}^{S}P_{i}^{2}\right)}^{-1}-1$$

where *P_i_* is the percentage of individuals belonging to species *i* and *S* is species richness.

### 4.4. Data Analysis

Statistical assumptions were verified using Shapiro–Wilk normality tests (*p* > 0.05) and Levene’s homogeneity of variance tests (*p* > 0.05). Data transformations were applied when necessary to meet assumptions. The effects of canopy litter treatment, stand density, time, and their interactions effects on forest canopy structure, light environment, and understory diversity indices were assessed using three-way ANOVA. Differences across density gradients were compared using one-way ANOVA with Duncan’s multiple range tests (*p* < 0.05). Treatment-specific variations were evaluated with independent-sample t-tests. Pearson correlation analysis was used to quantify relationships between canopy structure, light environment and understory vegetation parameters. Significant correlations (*p* < 0.05) were further examined using linear or nonlinear regression models. All statistical analyses were conducted using the SPSS Statistical Package (SPSS 29.0, IBM Corp., Armonk, NY, USA).

## 5. Conclusions

This study demonstrates that canopy litter removal in *C. lanceolata* plantations enhances understory light availability, which in turn regulates the balance between shade-tolerant and light-demanding species and accelerates successional trajectories in the short term. All these effects were density-dependent. However, whether this effect will alter the long-term potential dominance of fast-growing pioneer species and overall community stability, as well as its broader impacts on ecosystem functions such as carbon sequestration, nutrient cycling, and soil microbial activity, remains highly uncertain. Future research should therefore prioritize long-term monitoring of understory dynamics under varying stand densities, with particular emphasis on quantifying stand-level light use efficiency (LUE), carbon stocks, soil microbial functions, and overall stand productivity. Incorporating the effects of canopy litter removal into forest growth and biodiversity prediction models will enhance the predictive capacity and adaptability of management strategies for subtropical plantation under future climate change scenarios.

## Figures and Tables

**Table 1:** Three-way analysis of variance (ANOVA) of the effects of canopy treatment (df = 1), forest density (df = 5), time (df = 2), and their interactions on canopy structure in Chinese fir plantations.

Values	LAI		MTA	
	*F*	*P*	*F*	*P*
Treatment	125.36	**<0.001**	1.3	0.255
Density	1074.46	**<0.001**	257.05	**<0.001**
Time	647.84	**<0.001**	3838.22	**<0.001**
Treatment × density	2.16	0.060	3.59	**<0.01**
Treatment × Time	6.04	**<0.01**	9.51	**<0.001**
Density × Time	77.53	**<0.001**	52.76	**<0.001**
Treatment × density × Time	14.63	**<0.001**	8.25	**<0.001**

LAI leaf area index; MTA mean tilt angle of leaves.

**Table 2:** Three-way analysis of variance (ANOVA) of the effects of canopy treatment (df = 1), forest density (df = 5), time (df = 2), and their interactions on understory light in Chinese fir plantations.

Values	PPFD		T		R/B		R/FR	
	*F*	*P*	*F*	*P*	*F*	*P*	*F*	*P*
Treatment	859.28	**<0.001**	857.34	**<0.001**	0.14	0.144	0.22	0.222
Density	3704.44	**<0.001**	3446.56	**<0.001**	6.65	**<0.001**	14.64	**<0.001**
Time	9781.54	**<0.001**	128.63	**<0.001**	55.68	**<0.001**	11.99	**<0.001**
Treatment × density	13.98	**<0.001**	13.06	**<0.001**	2.28	**<0.05**	1.09	0.368
Treatment × time	14.96	**<0.001**	38.03	**<0.001**	1.19	0.305	2.95	0.055
Density × time	109.1	**<0.001**	94.45	**<0.001**	3.02	**<0.01**	5.15	**<0.001**
Treatment × density × Time	13.69	**<0.001**	10.69	**<0.001**	3.21	**<0.001**	3.25	**<0.001**

PPFD photosynthetic photon flux density, T light transmittance, R/B red:blue ratio, and R/FR red:far-red ratio.

**Table 3:** Effects of canopy litter removal and retention treatments on understory composition in Chinese fir plantations after 2, 4 and 5 years.

Composition	Years After Removal	Herb		Shrub		Total	
		Retention	Removal	Retention	Removal	Retention	Removal
Family	2	36	38	11	10	39	39
	4	32	37	7	12	33	38
	5	31	33	16	6	34	33
Genera	2	54	60	13	13	57	61
	4	48	59	9	14	50	60
	5	46	52	19	8	53	52
Species	2	65	67	13	14	78	81
	4	55	71	9	14	64	85
	5	53	63	20	9	73	72

**Table 4:** Three-way analysis of variance (ANOVA) of the effects of canopy treatment (df = 1), forest density (df = 1), time (df = 2), and their interactions on understory diversity in Chinese fir plantations.

Values	*D*		*H*		*J*		*OD*	
	*F*	*P*	*F*	*P*	*F*	*P*	*F*	*P*
Treatment	21.02	**<0.001**	52.13	**<0.001**	0.58	0.448	133.41	**<0.001**
Density	4.83	<0.001	12.11	**<0.001**	4.39	**<0.01**	11.24	**<0.001**
Time	3.11	0.051	2.16	0.123	56.49	**<0.001**	5.7	**<0.01**
Treatment × Density	0.89	0.495	5.26	**<0.001**	4.64	**<0.001**	1.53	0.190
Treatment × Time	4.64	**<0.01**	3.67	**<0.05**	6.83	**<0.01**	5.1	**<0.01**
Density × Time	1.7	0.097	3.37	**<0.01**	2.38	**<0.05**	4.21	**<0.01**
Treatment × Density × Time	1.6	0.123	0.96	0.488	2.49	**<0.05**	1.65	0.110

*D* Simpson index, *H* Shannon–Wiener index, *J* Pielou evenness, *OD* diversity of oddness index.

**Table 5:** The correlations between stand canopy structure indicators and light environment indicators.

Index	LAI	MTA	PPFD	T	R/B	R/FR
LAI	1	0.19 **	−0.59 **	−0.76 **	−0.30 **	0.31 **
MTA		1	0.35 **	−0.21 **	0.39 **	0.04
PPFD			1	0.77 **	0.48 **	0.23 **
T				1	0.25 **	0.20 **
R/B					1	0.68 **
R/FR						1

**, *p* < 0.01. See Table 2 and Table 3 for explanation of the abbreviations.

**Table 6:** Multivariate analysis of variance of the effects of overstory canopy structure, understory light on understory vegetation diversity.

Index	*D*	*H*	*J*	*OD*
PPFD	0.270 **	0.309 **	−0.015	0.282 **
T	0.216 *	0.337 **	−0.046	0.299 **
R/B	0.330 **	0.132	−0.064	0.265 **
R/FR	0.253 **	0.17	−0.157	0.265 **
LAI	−0.270 **	−0.377 **	0.275 **	−0.268 **
MTA	0.129	0.022	0.159	0.015

*, *p* < 0.05; **, *p* < 0.01. See Table 5 for explanation of the abbreviations.

**Table 7:** The basic situation of Chinese fir plantations with different densities before canopy litter removal in 2019.

Block Number	Stand Density (Stems·hm^−2^)	Latitude (N)	Longitude (E)	Slope and Aspect	Elevation (m a.s.l)	Mean DBH (cm)	Mean Height (m)
Ⅰ	1800 (M1)	26°9′30.60″	117°27′36.54″	17° NW	180.07	13.72	11.59
Ⅰ	2400 (M2)	26°9′31.38″	117°27′35.40″	28° SW	186.41	13.67	11.92
Ⅰ	3000 (M3)	26°9′25.80″	117°27′36.60″	20° SW	183.36	12.20	11.93
Ⅰ	3600 (M4)	26°9′34.02″	117°27′35.46″	24° SW	178.52	12.20	10.66
Ⅰ	4200 (M5)	26°9′35.04″	117°27′35.34″	28° SW	179.79	11.71	11.40
Ⅰ	4800 (M6)	26°9′31.86″	117°27′36.84″	19° SW	182.19	11.86	11.85
Ⅱ	1800 (M1)	26°9′33.12″	117°27′36.48″	17° NW	176.46	14.15	12.84
Ⅱ	2400 (M2)	26°9′31.14″	117°27′32.48″	23° SW	167.59	13.86	11.42
Ⅱ	3000 (M3)	26°9′27.35″	117°27′37.34″	24° NW	178.85	13.02	13.26
Ⅱ	3600 (M4)	26°9′31.98″	117°27′34.38″	20° NW	173.09	13.72	12.09
Ⅱ	4200 (M5)	26°9′30.30″	117°27′31.80″	22° SW	166.56	11.88	11.05
Ⅱ	4800 (M6)	26°9′31.62″	117°27′33.30″	22° SW	169.24	11.48	11.64
Ⅲ	1800 (M1)	26°9′31.44″	117°27′36.96″	24° W	178.14	14.52	13.59
Ⅲ	2400 (M2)	26°9′30.90″	117°27′35.34″	20° SW	187.23	15.31	13.67
Ⅲ	3000 (M3)	26°9′27.60″	117°27′36.90″	25° SW	182.24	13.87	12.94
Ⅲ	3600 (M4)	26°9′28.80″	117°27′36.78″	23° SW	183.48	12.40	11.46
Ⅲ	4200 (M5)	26°9′28.40″	117°27′36.72″	17° SW	181.52	11.99	11.78
Ⅲ	4800 (M6)	26°9′26.46″	117°27′36.66″	30° W	179.22	13.18	13.15

## Data Availability

All analysed data are presented in the main text and Supplementary Materials.

## Supplementary Materials

The following supporting information can be downloaded at https://www.mdpi.com/article/10.3390/plants14203144/s1, Table S1: Effects of canopy litter retention and removal on species richness of shrubs, herbs and total vegetation in different densities of Chinese fir plantations after 2, 4, and 5 years of treatments; Table S2: Understory composition and the top five dominant species between canopy litter retention and removal treatments at varied planting densities after 2 years (2021); Table S3: Understory composition and the top five dominant species between canopy litter retention and removal treatments at varied planting densities after 4 years (2023); Table S4: Understory composition and the top five dominant species between canopy litter retention and removal treatments at varied planting densities after 5 years (2024).

## References

[1] Payn T., Carnus J.M., Freer-Smith P., Kimberley M., Kollert W., Liu S., Orazio C., Rodriguez L., Silva L.N., Wingfield M.J. (2015). Changes in planted forests and future global implications. For. Ecol. Manag..

[2] FAO (2021). Global Forest Resources Assessment 2020: Main Report. Rome. https://www.fao.org/3/ca9825en/ca9825en.pdf.

[3] Yang Y.S., Wang L.X., Yang Z.J., Xu C., Xie J.S., Chen G.S., Lin C.F., Guo J.F., Liu X.F., Xiong D.C. (2018). Large ecosystem service benefits of assisted natural regeneration. J. Geophys. Res.-Biogeo..

[4] Zhou L.L., Cai L.P., He Z.M., Wang R.W., Wu P.F., Ma X.Q. (2016). Thinning increases understory diversity and biomass, and improves soil properties without decreasing growth of Chinese fir in southern China. Environ. Sci. Pollut. Res..

[5] Zhang H.M., Pan F.Y., Wen Z.M., Chen W.W., Zhou C.F. (2025). Impacts of successive Chinese fir plantations on soil carbon and nitrogen dynamics: Conclusive insights from metagenomic analysis. J. Environ. Manag..

[6] Yu X.T. (2000). Research progress of Chinese fir in 1990s. J. Fujian For. College.

[7] State Forestry Administration (SFA) of the People’s Republic of China (2014). Forest Resources in China (2014): The 8th National Forest Survey.

[8] Zhou L.L., Li S.B., Jia Y.Y., Heal K.V., He Z.M., Wu P.F., Ma X.Q. (2021). Spatiotemporal distribution of canopy litter and nutrient resorption in a chronosequence of different development stages of *Cunninghamia lanceolata* in southeast China. Sci. Total Environ..

[9] Zhang J.C., Sheng W.T. (2001). The study on decay of dead branches and leaves on living tress taken from crown into litter environment in a Chinese fir plantation, compared with decay in canopy. Sci. Silvae Sin..

[10] Liao Z.H., Fan S.H., Yu X.T. (1996). Study on the biomass among different-aged Chinse fir plantations at different site conditions. Ⅳ-Biomass of dead branches and leaves in the canopy. For. Res..

[11] Sheng W.T., Fan S.H. (2002). Impact of growth and development characters of Chinese fir and its plantation on the long-term site productivity. For. Res..

[12] Gao S.L., He Z.M., Huang Z.Q., Lin S.Z., Liu Z.M. (2015). Decomposition, carbon and nitrogen stable isotope and chemical composition of dead leaves clinging in a Chinese fir (*Cunninghamia lanceolata*) plantations. Chin. J. Ecol..

[13] Wu X.J., Li J.F., Jiang Y., Sun L.J., Wu P.F., Ma X.Q. (2024). Effect of simulated warming on nutrient release during the decomposition of leaf litter and canopy litter of Chinese fir based on displacement test. Acta Ecol. Sinca.

[14] Zhang Y.B., Duan B.L., Xian J.R., Korpelainen H., Li C.Y. (2011). Links between plant diversity, carbon stocks and environmental factors along a successional gradient in a subalpine coniferous forest in Southwest China. Forest Ecol. Manag..

[15] Méndez-Dewar G., González-Espinosa M., Equihua M. (2015). From seedling to sapling: Tree species responses to spatial and temporal understory light heterogeneity in disturbed tropical montane forests. Bot. Sci..

[16] Valerio M., Ibáñez R., Gazol A. (2021). The role of canopy cover dynamics over a decade of changes in the understory of an Atlantic Beech-Oak forest. Forests.

[17] Pelt R.V., Franklin J.F. (2000). Influence of canopy structure on the understory environment in tall, old-growth, conifer forests. Can. J. For. Res..

[18] Utsugi H., Araki M., Kawasaki T., Ishizuka M. (2006). Vertical distributions of leaf area and inclination angle, and their relationship in a 46-year-old *Chamaecyparis obtusa* stand. For. Ecol. Manag..

[19] Parker G.G. (2020). Tamm review: Leaf Area Index (LAI) is both a determinant and a consequence of important processes in vegetation canopies. For. Eco. Manag..

[20] Valiente-Banuet A., Verdú M., Valladares F., García-Fayos P. (2010). Functional and evolutionary correlations of steep leaf angles in the mexical shrubland. Oecologia.

[21] Yang X., Li R., Jablonski A., Stovall A., Kim J., Yi K., Ma Y.X., Beverly D., Phillips R., Novick K. (2023). Leaf angle as a leaf and canopy trait: Rejuvenating its role in ecology with new technology. Ecol. Lett..

[22] Valladares F., Skillman J.B., Pearcy R.W. (2002). Convergence in light capture efficiencies among tropical forest understory plants with contrasting crown architectures: A case of morphological compensation. Am. J. Bot..

[23] Plue J., Van Gils B., De Schrijver A., Peppler-Lisbach C., Verheyen K. (2013). Forest herb layer response to long-term light deficit along a forest developmental series. Acta Oecol..

[24] Brenes-Arguedas T., Roddy A.B., Coley P.D., Kuesar T.A. (2011). Do differences in understory light contribute to species distributions along a tropical rainfall gradient?. Oecologia.

[25] Dou H., Niu G., Gu M., Masabni J.G. (2017). Effects of light quality on growth and phytonutrient accumulation of herbs under controlled environments. Horticulturae.

[26] Jankowska-Blaszczuk M., Daws M.I. (2007). Impact of red: Far red ratios on germination of temperate forest herbs in relation to shade tolerance, seed mass and persistence in the soil. Funct. Ecol..

[27] Wan P., He R. (2020). Canopy structure and understory light characteristics of a natural *Quercus aliena var. acuteserrata* forest in China northwest: Influence of different forest management methods. Ecol. Eng..

[28] de Mattos E.M., Binkley D., Campoe O.C., Alvares C.A., Stape J.L. (2020). Variation in canopy structure, leaf area, light interception and light use efficiency among Eucalyptus clones. For. Ecol. Manag..

[29] Raabe K., Písek J., Sonnentag O., Annuk K. (2015). Variations of leaf inclination angle distribution with height over the growing season and light exposure for eight broadleaf tree species. Agr. Forest Meteorol..

[30] Navrátil M., Špunda V., Marková I., Janouš D. (2007). Spectral composition of photosynthetically active radiation penetrating into a Norway spruce canopy: The opposite dynamics of the blue/red spectral ratio during clear and overcast days. Trees.

[31] Leuchner M., Menzel A., Werner H. (2007). Quantifying the relationship between light quality and light availability at different phenological stages within a mature mixed forest. Agr. For. Meteorol..

[32] Barbier S., Gosselin F., Balandier P. (2008). Influence of tree species on understory vegetation diversity and mechanisms involved—A critical review for temperate and boreal forests. For. Ecol. Manag..

[33] Sims D.A., Gamon J.A. (2002). Relationships between leaf pigment content and spectral reflectance across a wide range of species, leaf structures and developmental stages. Remote Sens. Environ..

[34] McDowell N.G., Adams H.D., Bailey J.D., Kolb T.S. (2007). The role of stand density on growth efficiency, leaf area index, and resin flow in southwestern ponderosa pine forests. Can. J. For. Res..

[35] Akers M.K., Kane M., Zhao D.H., Teskey R.O., Daniels R.F. (2013). Effects of planting density and cultural intensity on stand and crown attributes of mid-rotation loblolly pine plantations. For. Eco. Manag..

[36] Farooq T.H., Yan W., Chen X.Y., Shakoor A., Rashid M.H.U., Gilani M.M., He Z.M., Wu P.F. (2020). Dynamics of canopy development of *Cunninghamia lanceolata* mid-age plantation in relation to foliar nitrogen and soil quality influenced by stand density. Glob. Ecol. Conserv..

[37] Valladares F., Niinemets Ü. (2008). Shade tolerance, a key plant feature of complex nature and consequences. Annu. Rev. Ecol. Evol. S..

[38] Brelsford C.C., Trasser M., Paris T., Hartikainen S.M., Robson M. (2022). Understory light quality affects leaf pigments and leaf phenology in different plant functional types. Physiol. Plantarum..

[39] Liu L.Q., Zhou G.Y., Zhao H.B., Li L., Qiu Z.J. (2013). Impact of simulated forest canopy damage on understory biodiversity of natural *Castanopsis fissa* community. For. Res..

[40] Crouzeilles R., Curran M., Ferreira M.S., Lindenmayer D.B., Grelle C.E.V., Rey Benayas J.M. (2016). A global meta-analysis on the ecological drivers of forest restoration success. Nat Commun..

[41] Skarpe C. (1990). Shrub layer dynamics under different herbivore densities in an arid savanna, Botswana. J. Appl. Ecol..

[42] Nicotra A.B., Chazdon R.L., Iriarte S.V.B. (1999). Spatial heterogeneity of light and woody seedling regeneration in tropical wet forests. Ecology.

[43] Majasalmi T., Rautiainen M. (2020). The impact of tree canopy structure on understory variation in a boreal forest. Forest. Ecol. Manag..

[44] Chen Y.M., Cao Y. (2014). Response of tree regeneration and understory plant species diversity to stand density in mature *Pinus tabulaeformis* plantations in the hilly area of the Loess Plateau, China. Ecol. Eng..

[45] Ali A., Dai D., Akhtar K., Teng M.J., Yan Z.G., Urbina-Cardona N., Mukkerova J., Zhou Z.X. (2019). Response of understory vegetation, tree regeneration, and soil quality to manipulated stand density in a *Pinus massoniana* plantation. Glob. Ecol. Conserv..

[46] Helbach J., Frey J., Messier C., Mörsdorf M., Scherer-Lorenzen M. (2022). Light heterogeneity affects understory plant species richness in temperate forests supporting the heterogeneity-diversity hypothesis. Ecol. Evol..

[47] Ampoorter E., Selvi F., Auge H., Lander B., Sigrid B., Elisa C., Andrea C., Mariangela F., Kalliopi R., Nurlaila S.N. (2016). Driving mechanisms of overstorey-understory diversity relationships in European forests. Perspect. Plant Ecol..

[48] Cazzolla Gatti R., Di Paola A., Bombelli A., Noce S., Valentini R. (2017). Exploring the relationship between canopy height and terrestrial plant diversity. Plant Ecol..

[49] Comita L.S., Queenborough S.A., Murphy S.J., Eck J.L., Xu K.Y., Krishnadas M., Beckman N., Zhu Y. (2014). Testing predictions of the Janzen-Connell hypothesis: A meta-analysis of experimental evidence for distance- and density-dependent seed and seedling survival. J. Ecol..

[50] Su S.C., Jin N.Q., Wei X.L. (2024). Effects of thinning on the understory light environment of different stands and the photosynthetic performance and growth of the reforestation species *Phoebe bournei*. J. For. Res..

[51] Farooq T.H., Wu W.J., Tigabu M., Ma X.Q., He Z.M., Rashid M.H.U., Gilani M.M., Wu P.F. (2019). Growth, biomass production and root development of Chinese fir in relation to initial planting density. Forests.

[52] Yirdaw E., Luukkanen O. (2004). Photosynthetically active radiation transmittance of forest plantation canopies in the Ethiopian highlands. For. Ecol. Manag..

[53] DeJong T.M. (1975). A comparison of three diversity indices based on their components of richness and evenness. Oikos.

[54] Kvålseth T.O. (1991). Note on biological diversity, evenness, and homogeneity measures. Oikos.

