# Supplementary material for: Effects of Canopy Litter Removal on Canopy Structure, Understory Light and Vegetation Dynamics in Cunninghamia lanceolata Plantations of Varying Densities
Source: Plants (Basel). 2025 Oct 12;14(20):3144. doi: 10.3390/plants14203144 (PMC12566832; doi:10.3390/plants14203144)
Supplement: Supplementary file 1 [file plants-14-03144-s001.zip › plants-3887948-supplementary.pdf]

Supplementary Material

Effects of Canopy Litter Removal on Canopy Structure, Understory Light and Vegetation Dynamics in *Cunninghamia lanceolata* Plantations of Varying Densities

Lili Zhou[1], Lixian Zhang[2], Qi Liu[3], Yulong Chen[4], Zongming He[3], Shubin Li[3], Xiangqing Ma[3*]

[1]*College of Geography and Oceanography, Minjiang University, Fuzhou 350108, China*

[2]*Research Institute of Subtropical Forestry, Chinese Academy of Forestry, Hangzhou 31140, China*

[3]*Forestry College, Fujian Agriculture and Forestry University, Fuzhou 350002, China*

[4]*College of Resources and Environment, Fujian Agriculture and Forestry University, Fuzhou 350002 China*

**Correspondence: lxymxq@126.com*

Number of pages: **5**, Number of tables: **4**

Table S1 Effects of canopy litter retention and removal on species richness of shrubs, herbs and total vegetation in different densities of Chinese fir plantations after 2, 4, and 5 years of treatments.

Densities	Layer	2nd year		4th year		5th year	
		Retention	Removal	Retention	Removal	Retention	Removal
M1	Shrub	6	6	4	6	4	6
	Herb	29	29	26	59	33	37
	Total	35	35	30	65	37	43
M2	Shrub	4	2	5	2	9	4
	Herb	34	32	29	66	29	39
	Total	38	34	34	68	38	43
M3	Shrub	5	8	2	4	5	3
	Herb	31	30	33	72	28	36
	Total	36	38	35	76	33	39
M4	Shrub	2	0	5	2	3	4
	Herb	32	32	35	62	31	33
	Total	34	32	40	64	34	37
M5	Shrub	2	2	2	3	2	1
	Herb	42	37	24	56	27	31
	Total	44	39	26	59	29	32
M6	Shrub	0	0	2	3	3	2
	Herb	25	25	21	50	21	26
	Total	25	25	23	53	24	28

Table S2 Understory composition and the top five dominant species between canopy litter retention and removal treatments at varied planting densities after 2 years (2021). The species are listed by decreasing order of important value index (IVI) and the values in brackets represent IVI.

Stand density (stems • hm^{-2})	Species, Genes and Family		Dominant species in shrub and herb layer, respectively	
	Retention	Removal	Retention	Removal
1800 (M1)	6, 6 and 5 in shrub layer<br>29, 25 and 18 in herb layer	6, 6 and 5 in shrub layer<br>29, 27 and 25 in herb layer	*I. tessellatus* (52.42), *I. pubescens* (17.76), *T. mollissima* (10.22), *P. amarus* (7.89), *C. aculeatus* (6.11) in shrub layer; *W. japonica* (18.44), *L. orbiculata* (15.15), *A. metteniana* (7.54), *D. fuscipes* (5.38), *M. dodecandrum* (4.51) in herb layer	*I. tessellatus* (48.37), *P. amarus* (30.25), *I. pubescens* (6), *E. nitida* (5.37), *S. laurina* (5.3) in shrub layer; *W. japonica* (27.19), *L. orbiculata* (14.93), *A. metteniana* (6.87), *D. fuscipes* (4.84), *D. pedata* (4.13) in herb layer
2400 (M2)	4, 4 and 4 in shrub layer<br>34, 31 and 26 in herb layer	2, 2 and 2 in shrub layer<br>32, 27 and 24 in herb layer	*S. sumuntia* (33.1), *S. faberi* (25.22), *K. longipedunculata* (22.1), *V. carlesii* (19.58) in shrub layer; *W. japonica* (21.1), *A. metteniana* (16.3), *L. orbiculata* (10.09), *D. fuscipes* (6.73), *D. dilatatum* (4) in herb layer	*L. glaber*(69.51), *C. kochiana*(30.49) in shrub layer; *A. metteniana* (18.34), *W. japonica* (14.15), *L. orbiculata* (8.47), *D. fuscipes* (7.01), *P. semipinnata* (5.83) in herb layer
3000 (M3)	5, 5 and 3 in shrub layer<br>31, 25 and 20 in herb layer	8, 8 and 7 in shrub layer<br>30, 26 and 19 in herb layer	*E. nitida* (32.72), *P. amarus* (30.06), *A. millettii* (14.75), *S. lanceifolia* (13.29), *I. tessellatus* (9.18) in shrub layer; *W. japonica* (12.93), *L. orbiculata* (12.14), *D. fuscipes* (8.68), *A. metteniana* (8.36), *A. dilatata* (4.42) in herb layer	*I. tessellatus* (24.14), *I. pubescens* (15.44), *S. superba* (13.3), *M. pubescens* (13.05), *M. grijsii* (10.51) in shrub layer; *L. orbiculata* (17.61), *W. japonica* (13.68), *D. fuscipes* (7.94), *L. gracile* (6.53), *A. metteniana* (6.24) in herb layer
3600 (M4)	2, 2 and 2 in shrub layer<br>32, 27 and 20 in herb layer	0, 0 and 0 in shrub layer<br>32, 28 and 19 in herb layer	*I. pubescens* (51.32), *P. amarus* (48.68) in shrub layer; *A. metteniana* (12.27), *L. orbiculata* (11.82), *W. japonica* (10.61), *D. dilatatum* (7.44), *D. fuscipes* (6.49) in herb layer	None in shrub layer; *W. japonica* (13.94), *L. orbiculata* (11.58), *A. metteniana* (9.74), *D. fuscipes* (8.35), *M. strigosa* (7.29) in herb layer
4200 (M5)	2, 2 and 2 in shrub layer<br>42, 34, and 26 in herb layer	2, 2 and 2 in shrub layer<br>37, 33 and 23 in herb layer	*I. pubescens* (58.33), *S. sumuntia* (41.67) in shrub layer; *W. japonica* (15.95), *L. orbiculata* (12.5), *A. metteniana* (8.12), *C. barometz* (4.85), *D. sparsa* (4.64) in herb layer	*I. pubescens* (54.33), *S. sumuntia* (45.67) in shrub layer; *W. japonica* (18.1), *L. orbiculata* (16.25), *A. metteniana* (11.45), *D. dilatatum* (6.84), *D. sparsa* (4.25) in herb layer
4800 (M6)	0, 0 and 0 in shrub layer<br>25, 21 and 19 in herb layer	0, 0 and 0 in shrub layer<br>25, 22 and 18 in herb layer	None in shrub layer; *A. metteniana* (20.98), *L. orbiculata* (13.86), *W. japonica* (7.7), *A. hispidus* (6.64), *D. fuscipes*(5.99) in herb layer	None in shrub layer; *L. orbiculata* (21.23), *A. metteniana* (14.83), *W. japonica* (10), *L. gracile* (6.15), *D. fuscipes* (3.33) in herb layer

Table S3 Understory composition and the top five dominant species between canopy litter retention and removal treatments at varied planting densities after 4 years (2023). The species are listed by decreasing order of important value index (IVI) and the values in brackets represent IVI.

Stand density (stems • hm^{-2})	Species, Genes and Family		Dominant species in shrub and herb layer, respectively	
	Retention	Removal	Retention	Removal
1800 (M1)	4, 4 and 3 in shrub layer<br>26, 24 and 17 in herb layer	6, 6 and 5 in shrub layer<br>59, 38 and 26 in herb layer	*I. tessellatus* (73.33), *P. amarus* (11.42), *M. japonica* (7.82), *E. nitida* (7.43) in shrub layer;<br>*L. gracile* (19.85), *W. japonica* (16.76), *A. metteniana* (8.02), *L. orbiculata* (6.72), *D. pedata* (5.98) in herb layer	*I. tessellatus* (51.82), *M. japonica* (16.37), *S. superba* (11.87), *I. pubescens* (7.41), *P. amarus* (7.04) in shrub layer;<br>*W. japonica* (20.1), *L. gracile* (8.7), *A. metteniana* (8.03), *L. orbiculata* (5.52), *C. patens* (4.95) in herb layer
2400 (M2)	5, 5 and 3 in shrub layer<br>29, 27 and 20 in herb layer	2, 2 and 2 in shrub layer<br>66, 40 and 26 in herb layer	*A. millettii* (37.89), *I. tessellatus* (26.24), *E. nitida* (13.85), *P. amarus* (13.58), *R. columellaris* (8.45) in shrub layer;<br>*W. japonica* (14.66), *A. metteniana* (8.99), *P. dispar* (8.64), *O. undulatifolius* (7.16), *L. gracile* (4.65) in herb layer	*I. tessellatus* (72.03), *C. kawakamii* (27.98) in shrub layer;<br>*W. japonica* (15.83), *A. metteniana* (14.56), *L. gracile* (9.13), *P. dispar* (5.17), *L. orbiculata* (4.67) in herb layer
3000 (M3)	2, 2 and 1 in shrub layer<br>33, 29 and 23 in herb layer	4, 4 and 3 in shrub layer<br>72, 41 and 27 in herb layer	*I. tessellatus* (56.17), *P. amarus* (43.83) in shrub layer;<br>*P. dispar* (12.51), *W. japonica* (11.71), *L. gracile* (7.99), *L. orbiculata* (5.4), *D. fuscipes* (5.3) in herb layer	*I. tessellatus* (68), *C. kochiana* (13.46), *S. sumuntia* (9.92), *P. amarus* (8.62) in shrub layer;<br>*W. japonica* (11.39), *L. gracile* (10.89), *A. metteniana* (9.76), *C. patens* (7.82), *P. dispar* (6.93) in herb layer
3600 (M4)	5, 5, and 5 in shrub layer<br>35, 30, and 25 in herb layer	2, 2, and 2 in shrub layer<br>62, 39, and 30 in herb layer	*I. tessellatus* (41.04), *A. millettii* (17.79), *S. laurina* (17.58), *R. columellaris* (13.23), *L. communis* (10.36) in shrub layer;<br>*W. japonica* (17.6), *L. gracile* (15.79), *P. dispar* (10.68), *D. fuscipes* (7.73), *L. orbiculata* (4.79) in herb layer	*I. pubescens* (55.99), *S. lanceifolia* (44.02) in shrub layer<br>*L. gracile* (21.28), *P. dispar* (13.65), *W. japonica* (9.03), *D. fuscipes* (8.27), *A. metteniana*(7.43) in herb layer
4200 (M5)	2, 2 and 2 in shrub layer<br>24, 20 and 18 in herb layer	3, 3 and 3 in shrub layer<br>56, 33 and 29 in herb layer	*M. japonica* (71.27), *P. amarus* (28.73) in shrub layer;<br>*L. gracile* (13.29), *A. metteniana* (10.54), *W. japonica* (9.61), *O. undulatifolius* (9.51), *P. dispar* (8.19) in herb layer	*I. tessellatus* (48.92), *A. lindleyana* (32.3), *S. grijsii* (18.78) in shrub layer;<br>*L. gracile* (13.38), *A. metteniana* (13.18), *C. patens* (10.82), *W. japonica* (10.49), *L. orbiculata* (8.2) in herb layer
4800 (M6)	2, 2 and 2 in shrub layer<br>21, 18 and 16 in herb layer	3, 3 and 3 in shrub layer<br>50, 33 and 24 in herb layer	*C. kochiana* (52.05), *P. amarus* (47.95) in shrub layer;<br>*L. gracile* (21.42), *A. metteniana* (13.47), *C. patens* (12.32), *W. japonica* (7.65), *L. orbiculata* (7.44) in herb layer	*I. tessellatus* (65.67), *A. sinica* (17.98), *P. microphylla* (16.35) in shrub layer<br>*P. dispar* (15.66), *M. hancei* (11.75), *A. metteniana* (8.18), *D. dilatatum* (6.91), *L. gracile* (6.9) in herb layer

Table S4 Understory composition and the top five dominant species between canopy litter retention and removal treatments at varied planting densities after 5 years (2024). The species are listed by decreasing order of important value index (IVI) and the values in brackets represent IVI.

Stand density (stems • hm^{-2})	Species, Genes and Family		Dominant species in shrub and herb layer, respectively	
	Retention	Removal	Retention	Removal
1800 (M1)	4, 4 and 4 in shrub layer<br>33, 29 and 21 in herb layer	6, 6 and 5 in shrub layer<br>37, 32 and 24 in herb layer	*P. amarus* (57.66), *P. glaucus* (21.45), *F. hirta* (11.82), *C. oleifera* (9.18) in shrub layer;<br>*W. japonica* (12.67), *O. undulatifolius* (12), *L. gracile* (9.9), *A. metteniana* (8.88), *D. fuscipes* (5.93) in herb layer	*I. tessellatus* (48.1), *P. amarus* (18.3), *M. japonica* (10.78), *M. velutina* (9.97), *A. millettii* (6.92) in shrub layer;<br>*W. japonica* (9.97), *D. dilatatum* (8.91), *A. metteniana* (8.5), *O. undulatifolius* (6.58), *A. flabellulatum* (5.59) in herb layer
2400 (M2)	9, 9 and 8 in shrub layer<br>29, 26 and 22 in herb layer	4, 4 and 4 in shrub layer<br>39,35 and 27 in herb layer	*P. amarus* (43.87), *S. superba* (9.46), *M. velutina* (9.39), *D. dubia* (9.3), *A. lindleyana* (7.33) in shrub layer;<br>*W. japonica* (12.73), *A. metteniana* (9.14), *D. dilatatum* (8.97), *P. dispar* (7.71), *M. hancei* (7.39) in herb layer	*P. amarus* (56.57), *M. japonica* (15.28), *M. grijsii* (14.27), *E. nitida* (13.89) in shrub layer;<br>*W. japonica* (12.05), *L. gracile* (10.59), *A. metteniana* (10.03), *D. fuscipes* (6.08), *P. dispar Kunze* (4.42) in herb layer
3000 (M3)	5, 5 and 4 in shrub layer<br>28, 24 and 17 in herb layer	3, 3 and 2 in shrub layer<br>36, 31 and 25 in herb layer	*P. amarus* (49.59), *I. tessellatus* (21.97), *C. carlesii* (12.24), *I. pubescens* (9.21), *V. montana* (6.98) in shrub layer;<br>*W. japonica* (12.05)，*L. gracile* (10.16) ，*A. metteniana* (9.12)，*O. undulatifolius* (8.35)，*P. dispar* (6.15) in herb layer	*P. amarus* (45.47), *I. tessellatus* (34.95), *S. buxifolium* (19.58) in shrub layer;<br>*W. japonica* (14.79), *O. undulatifolius* (7.88), *L. gracile* (7.82), *A. metteniana* (6.99), *M. hancei Prantl* (4.8) in herb layer
3600 (M4)	3, 3 and 3 in shrub layer<br>31, 28 and 21 in herb layer	4, 4 and 3 in shrub layer<br>33, 28 and 21 in herb layer	*P. amarus* (49.38), *S. buxifolium* (28.51), *I. pubescens* (22.11) in srhub layer;<br>*L. gracile* (11.58), *W. japonica* (11.55), *O. undulatifolius* (9.86), *M. hancei*(8.23), *D. dilatatum* (8.17) in herb layer	*P. amarus* (59.16), *E. nitida* (18.58), *M. japonica* (13.3), *I. tessellatus* (8.97) in shrub layer<br>*L. gracile* (11.8)，*D. dilatatum* (8.93)，*W. japonica* (8.68), *O. undulatifolius* (8.16), *M. hancei* (6.96) in heb layer
4200 (M5)	2, 2 and 2 in shrub layer<br>27, 24 and 18 in herb layer	1, 1 and 1 in shrub layer<br>31, 28 and 19 in herb layer	*E. nitida* (64.49), *A. grossedentata* (35.51) in shrub layer;<br>*D. dilatatum* (15.57), *L. gracile* (13.44), *W. japonica* (10.98), *M. hancei* (6.27), *A. metteniana* (6.21) in herb layer	*P. amarus* (100) in shrub layer;<br>*L. gracile* (19.64), *W. japonica* (11.71), *O. undulatifolius* (11.08), *A. metteniana* (6.42), *P. semipinnata* (5.59) in herb layer;
4800 (M6)	3, 3 and 3 in shrub layer<br>21, 19 and 16 in herb layer	2, 2 and 2 in shrub layer<br>26, 22 and 17 in herb layer	*C. kochiana* (42.39), *P. amarus* (29.1), *R. reflexus* (28.51) in shrub layer;<br>*L. gracile* (21.14), *M. hancei* (9.56), *P. semipinnata* (8.33), *W. japonica* (8.25), *A. metteniana* (7.37) in herb layer	*P. amarus* (68.73), *M. japonica* (31.27) in shrub layer;<br>*P. dispar* (17.13), *L. gracile* (16.96), *M. hancei* (10.39), *O. undulatifolius* (9.79), *A. metteniana* (7.7) in herb layer
